# Pest defences under weak selection exert a limited influence on the evolution of height growth and drought avoidance in marginal pine populations

**DOI:** 10.1098/rspb.2022.1034

**Published:** 2022-09-14

**Authors:** Yang Liu, Nadir Erbilgin, Blaise Ratcliffe, Jennifer G. Klutsch, Xiaojing Wei, Aziz Ullah, Eduardo Pablo Cappa, Charles Chen, Barb R. Thomas, Yousry A. El-Kassaby

**Affiliations:** ^1^ Department of Forest and Conservation Sciences, University of British Columbia, 2424 Main Mall, Vancouver, British Columbia V6T 1Z4, Canada; ^2^ McDonald Institute for Archaeological Research, University of Cambridge, Downing Street, Cambridge CB2 3DZ, UK; ^3^ Wolfson College, University of Cambridge, Barton Road, Cambridge CB3 9BB, UK; ^4^ Department of Renewable Resources, University of Alberta, 442 Earth Sciences Building, Edmonton, Alberta T6G 2E3, Canada; ^5^ Instituto Nacional de Tecnología Agropecuaria (INTA), Instituto de Recursos Biológicos, Centro de Investigación en Recursos Naturales, De Los Reseros y Doctor Nicolás Repetto s/n, 1686, Hurlingham, Buenos Aires, Argentina; ^6^ Consejo Nacional de Investigaciones Científicas y Técnicas (CONICET), Buenos Aires, Argentina; ^7^ Department of Biochemistry and Molecular Biology, 246 Noble Research Center, Oklahoma State University, Stillwater, OK 74078, USA

**Keywords:** climate change, common-garden approach, drought, forest pests, trait interactions, *Pinus contorta*

## Abstract

While droughts, intensified by climate change, have been affecting forests worldwide, pest epidemics are a major source of uncertainty for assessing drought impacts on forest trees. Thus far, little information has documented the adaptability and evolvability of traits related to drought and pests simultaneously. We conducted common-garden experiments to investigate how several phenotypic traits (i.e. height growth, drought avoidance based on water-use efficiency inferred from *δ*^13^C and pest resistance based on defence traits) interact in five mature lodgepole pine populations established in four progeny trials in western Canada. The relevance of interpopulation variation in climate sensitivity highlighted that seed-source warm populations had greater adaptive capability than cold populations. In test sites, warming generated taller trees with higher *δ*^13^C and increased the evolutionary potential of height growth and *δ*^13^C across populations. We found, however, no pronounced gradient in defences and their evolutionary potential along populations or test sites. Response to selection was weak in defences across test sites, but high for height growth particularly at warm test sites. Response to the selection of *δ*^13^C varied depending on its selective strength relative to height growth. We conclude that warming could promote the adaptability and evolvability of growth response and drought avoidance with a limited evolutionary influence from pest (biotic) pressures.

## 1. Introduction

Forests of boreal and temperate regions are dominated by gymnosperm trees in which conifers are a key component and comprise greater than 39% of the global forests [1]. In this era of unprecedented climate change, numerous studies have documented maladaptation of some tree species to environments due to adaptive constraints (e.g. long lifespans and slow migration rates) [2–4]. Coniferous trees show strong resiliency even to extreme climates [5], implying a high degree of adaptability to heterogeneous environments. However, global warming has lowered such resiliency by increasing the duration and frequency of natural disturbances including drought and insect outbreaks [6–11]. Drought could limit tree growth, which could further adversely affect resource allocation to tree defences against biotic agents [12–15]. The interacting effects of drought and insect attacks promote tree death possibly through depletion of carbohydrates and carbon-dependent defence metabolites [14,16–18].

Pines are considered drought-tolerant species and have well-defined defences against a broad range of ecologically and economically important insect herbivores and pathogens [7,11,19–21]. Frequent climatic events under ongoing global change such as protracted drought can impose an additional selective pressure on or directly affect functional traits that enable local adaptation to dry conditions [17]. To withstand drought stress, plants have evolved a drought avoidance strategy [22,23] involving reduced water loss through changes in hydraulic conductance to enhance water-use efficiency, and maintain cellular homeostasis during drought. Measurements of ecophysiological status can be used for determining water-use efficiency, such as carbon isotope discrimination *δ*^13^C [24]. High water-use efficiency inferred from *δ*^13^C indicates the potential to maximize survival under drought and thus has a synergistic effect on plant growth (fitness). On the other hand, effects of biotic interactions are, however, less predictable due in part to the specificity, conditionality and complexity of their relationship with many other factors [7]. For example, pest outbreaks have been promoted by direct effects of warmer temperatures on pest life cycles [25] and indirect effects of drought on improving host susceptibility by reducing the efficiency of tree defences [14,26–29]. Variable factors affecting host susceptibility to pests prompt its equivocal relationship with growth or drought avoidance. Currently, the interactions between multiple traits in pine populations are poorly understood.

**Figure 1.**
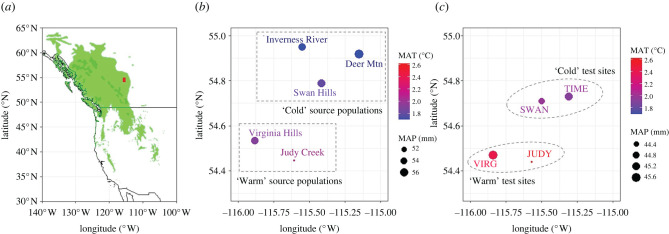
Map of the distribution range of *Pinus contorta* (*a*), five study populations (*b*) and four progeny trial test sites (*c*). The *Pinus contorta* distribution range is shaded in green on the map with our study region marked by a red rectangle. MAT: mean annual temperature; MAP: mean annual precipitation (monthly average). The study region is boreal forests, characteristic of a dry continental climate with cold winters and warm summers. Based on MAT, we defined: (i) JUDY and VIRG are ‘warm’ test sites, and TIME and SWAN are ‘cold’ test sites; and (ii) Judy Creek and Virginia Hills are ‘warm’ populations, and Deer Mtn, Inverness River and Swan Hills are ‘cold’ populations. In addition, four capital letters were used for test sites, and full site names denoted populations throughout the paper.

Range edge plant populations take on urgency for research, given that they are a more sensitive harbinger of climate change than central populations and may be trailing, suffering from declining population sizes and low genetic diversity, and thus at greater risk of mortality or extirpation [30]. In this study, we selected autochthonous populations of lodgepole pine (*Pinus contorta* Dougl. ex. Loud. var *latifolia* Englm.) located along the eastern edge of the species distribution range and relocated to four progeny trials (figure 1*a–c*) as analogues for future climate change scenarios (e.g. +1–2 °C). Our goal was to examine the adaptability and evolvability of several phenotypic traits in a multi-variate context, including height growth, *δ*^13^C indicating the ability to evade drought-caused physiological stress (i.e. drought avoidance), and host suitability to two most abundant pest species. Western gall rust (WGR; *Endocronartium harknessii* Hirats.) is an important fungal disease on lodgepole pine and widespread across the study region; the second pest is mountain pine beetle (MPB; *Dendroctonus ponderosae* Hopkins), which is one of the most important agents of lodgepole pine mortality in western North America (e.g. [7,25]). We expected that warming likely promotes both adaptive capacity and evolutionary potential for populations from a high latitude growing in proximal locations. To that end, we sought specifically to test for the following three hypotheses:

H1: Warming promotes tree growth and *δ*^13^C increase. Warm-origin populations growing in a warm test site have greater height and higher *δ*^13^C than cold populations in any test site; warm test sites create a higher evolutionary potential for height growth and *δ*^13^C than cold test sites.
H2: There is an indeterministic association between warm population and high pest susceptibility; evolutionary potential of pest susceptibility in warm versus cold test sites is not pronounced either.
H3: If H1 and H2 hold, then in a multi-dimensional trait space, a warm climate still generates higher evolutionary response to the selection of tree growth and *δ*^13^C, whereas response to selection in pest susceptibility remains weak and varies greatly within warm test sites.

## 2. Methods

### (a) Plant material and experimental design

We selected five lodgepole pine provenances (populations hereafter), representing a total of 224 maternal half-sib families, grown in four progeny test sites (greater than 35 years) arrayed along various climatic gradients in central Alberta, Canada (53–59 families from each test site used for this study; figure 1*b,c*; electronic supplementary material, table S1). All 224 families were divided into 21 sets, each consisted of about 12 families (electronic supplementary material, figure S1). At each site, the field design was sets nested in five replicates with 21 sets per replicate, and families within each set were planted in four-tree row plots at a 2.5 m × 2.5 m spacing. All sites were fenced and each trial had a border row of trees around the outside. Across the four progeny test sites, we chose a total of 1490 trees for phenotyping.

### (b) Phenotypic measurements

Detailed phenotypic trait measurement procedures were described in the electronic supplementary material, methods S1. Concisely, height growth (m) was measured at age 35 years with a clinometer. Carbon isotope ratio (*δ*^13^C, in ‰) analysis was performed at Alberta Innovates in Victoria, using outside slabs cut and ground from the 5 mm increment cores taken from the north side of each tree at approximately breast height (1.3 m) at age 35. Samples were analysed using an established method on a MAT253 Mass Spectrometer with Conflo IV interface (Thermo Fisher Scientific, Waltham, MA, USA) and a Fisons NA1500 EA (Fisons Instruments, Milan, Italy). We assessed the severity of WGR infection in the test sites by a qualitative scoring system with discrete categories ranging from no gall symptoms to deceased (four tiers) for all trees sampled at age 36. We also investigated these trees' suitability to MPB. Host tree suitability to MPB was evaluated by quantifying defence chemicals (mainly monoterpenes) using a gas chromatography/flame ionization detector (Agilent Tech., Santa Clara, CA, USA) based on cambial tissues collected by a hole punch when trees were actively growing, coinciding with MPB flight in western Canada. Then, chemical profiling was performed to test against MPB performance based on laboratory bioassays reported by Ullah *et al*. [31]. We used a cutoff of four categories to classify trees with different MPB suitability levels (see electronic supplementary material, methods S1 for details).

### (c) Data analysis

#### (i) Detrending phenotype

Based on raw phenotypic data, we detrended phenotypic traits caused by environmental variation within test site. We analysed each trait in each test site using a mixed model with a spatial autocorrelation. In the model, population was a fixed effect, and the random-effect terms consisted of replicate, set and genetic effects derived from pedigree information (details in electronic supplementary material, methods S2). The residuals included spatially dependent and independent components with a first-order autoregressive (co)variance structure (AR1 × AR1). The detrended phenotypic traits were obtained for each tree at each site by removing the estimated design effects and autoregressive residual effects. The detrended traits were used for all subsequent analyses unless otherwise indicated.

#### (ii) Phenotypic selection, evolutionary potential and response to selection

We performed selection analysis, as previously described [32], to estimate how natural selection operates on *δ*^13^C and pest suitability after adjusting for trait correlations. Succinctly, we used height as a surrogate for fitness, because fast growth leads to larger trees with higher survivorship and likely produces more offspring over a lifetime owing to a larger crown size. Following the Lande & Arnold (1983) method (formulae 4, 6c, 13b, and 14a [33]), we calculated for each test site and all sites pooled linear selection differentials (*s* = Cov[*w*, *z*]), linear selection gradients (*β* = *P*^−1^*s*), quadratic selection differentials $\left(C=\mathrm{Cov}[w,(z\;-\;\bar{z}){\left(z\;-\;\bar{z}\right)}^{T}]\right)$ and quadratic selection gradients (*γ* = *P*^−1^ *C P*^−1^), where *w* is the vector of relative fitness, *z* is the vector of phenotype, *P* is the phenotypic variance-covariance matrix of phenotypes (i.e. *G*-matrix). In all cases, the significance was determined by assessing statistical uncertainties using 5000 bootstrap replicates.

As natural selection acts on variation in phenotypes regardless of their genetic basis, we furthermore tested for genetic variation and evolvability with recourse to principles of heredity and evolution. We calculated the phenotype-based narrow-sense heritability by applying the formula, *h*^2^ = *V*_A_/(*V*_A_ + *V*_ɛ_), to each sample of the posterior distributions (model in the electronic supplementary material, Methods S3). The 95% highest posterior density intervals for the posterior distribution were used to capture the uncertainty in the *h*^2^ estimation. In addition to *h*^2^, the additive genetic coefficient of variance (C*V*_A_) is another commonly used measure of the evolvability of a given trait, as it links the trait to fitness and provides an estimate of the expected response to selection [34,35]. C*V*_A_ was calculated as *V*_A_ standardized by the trait mean (detrended then scaled), where *V*_A_ is extracted from the posterior distributions (model in electronic supplementary material, Methods S3).

While these univariate heritable variation parameters act mainly as an efficiency filter of inheritance across generations, the structure of genetic covariance among traits also affects evolutionary changes. Considering the intricacies of evolutionary dynamics, we used the posterior mean *G*-matrix across the iterations of each model to predict the evolutionary response to selection (**Δ*Z***) using the multi-variate breeder's equation [36],

2.1 $$\Delta Z=G\beta =\;\overset{n}{\underset{i=1}{\sum }}\lambda_{i}v_{i}v_{i}^{T}\beta ,$$

where given a set of *n* traits, ***λ*_i_** is an eigenvalue of additive genetic (co)variance matrix ***G***, ***v*_i_** is its corresponding eigenvectors and ***β*** is directional selection gradients for the traits (table 1). Given height as a proxy for fitness, we were unable to calculate the selection gradient for height. Instead, we used two sets of ***β*** values for height: 50% lower or higher than the selection gradient in *δ*^13^C, which equalled range limits between ***β*_[_***_δ_*_13C]_*0.5 and ***β*_[_***_δ_*_13C**]**_ for one set while the other set was between ***β*_[_***_δ_*_13C**]**_ and ***β*_[_***_δ_*_13C]_*1.5. Each ***β*** set was generated by increasing values within its limit for 100 steps at equal intervals. We calculated the 95% CIs based on the mean posterior distributions of **Δ*Z*** given different ***β*** values for height to capture the uncertainty in the **Δ*Z*** estimation. If 95% CIs did not overlap between pairwise comparisons, we took this as evidence that the trait underwent different evolutionary shifts between sites.

**Table 1.** Linear and quadratic selection gradients (*β* and *γ*) and selection differentials (*s* and *C*) for each focal trait in each or all progeny test sites of *Pinus contorta*. Height was used as a proxy for fitness and thus it was not possible to perform selection analysis for it. The signs and magnitudes indicate the direction and strength of linear (selection gradient *β* or selection differential *s*) or quadratic selection (selection gradient *γ* or selection differential *C*) on each trait in each or all test sites combined. Linear (directional) selection includes positive (i.e. genetic hitchhiking) and negative (i.e. background selection) selection. For quadratic selection, a negative significant selection value of *γ* or *C* indicates stabilizing selection, whereas a positive significant value is evidence for disruptive selection. Mean (s.e.) values were estimated and significance was determined by performing 5000 bootstrap samples. Significance: ***p < 0.0001, **p < 0.01, *p < 0.05.

trait	test site	linear selection (negative or positive)		quadratic selection (stabilizing or divergent)	
		*β*	*s*	*γ*	*C*
drought avoidance (*δ*^13^C)	TIME	0.020 (0.004)***	0.021 (0.004)***	−0.006 (0.006)	−0.006 (0.006)
	SWAN	0.023 (0.004)***	0.023 (0.004)***	−0.002 (0.006)	−0.002 (0.006)
	VIRG	0.002 (0.005)	0.002 (0.005)	−0.008 (0.007)	−0.008 (0.007)
	JUDY	0.010 (0.006)*	0.009 (0.006)	−0.016 (0.008)*	−0.015 (0.007)*
	all sites	0.029 (0.003)***	0.029 (0.002)***	−0.006 (0.004)	−0.005 (0.004)
severity of WGR	TIME	−0.009 (0.005)*	−0.009 (0.005)*	−0.008 (0.007)	−0.008 (0.006)
	SWAN	−0.004 (0.004)	−0.002 (0.004)	−0.009 (0.005)*	−0.008 (0.005)
	VIRG	−0.003 (0.005)	−0.003 (0.005)	−0.003 (0.006)	−0.003 (0.006)
	JUDY	−0.003 (0.005)	−0.003 (0.005)	−0.002 (0.005)	−0.003 (0.005)
	all sites	−0.002 (0.003)	−0.002 (0.002)	−0.003 (0.003)	−0.003 (0.003)
suitability to MPB	TIME	−0.002 (0.004)	−0.001 (0.004)	−0.001 (0.004)	0 (0.004)
	SWAN	−0.005 (0.004)	−0.004 (0.004)	−0.008 (0.004)*	−0.007 (0.004)*
	VIRG	−0.003 (0.005)	−0.003 (0.005)	−0.003 (0.005)	−0.002 (0.005)
	JUDY	−0.006 (0.005)	−0.005 (0.005)	−0.007 (0.004)*	−0.005 (0.004)
	all sites	−0.004 (0.002)*	−0.003 (0.002)	−0.004 (0.002)*	−0.004 (0.002)

## 3. Results

### (a) Correlative patterns in traits and trait-climate

**Figure 2.**
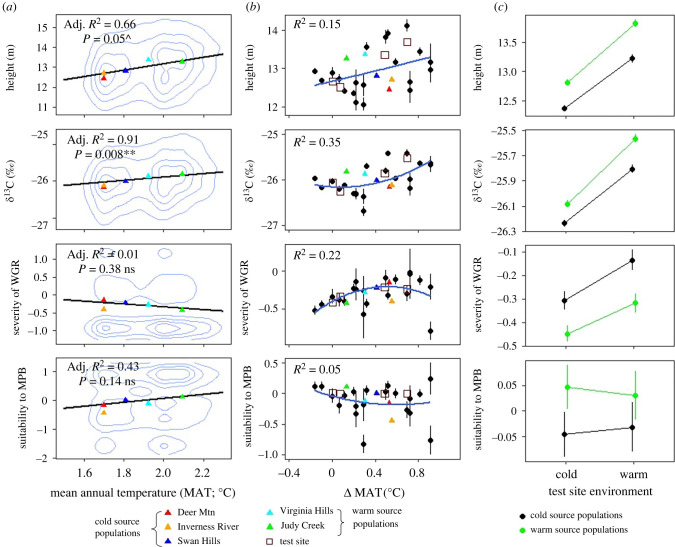
Population trait means as a function of MAT at site-of-origin, population differentiation for focal traits as a function of MAT transfer distance and mean trait values for each combination of the source population and test site groups. (*a*) Black lines depict a linear model-predicted relationship with 95% CI on a population basis. Significant relationships suggest local adaptation. The relative density of underlying data points is represented by contour lines. The trait values averaged by population are shown in coloured triangles. WGR and MPB denote western gall rust (*Endocronartium harknessii*) and mountain pine beetle (*Dendroctonus ponderosae*), respectively; both traits were scaled and high/low values are indicative of high/low pest symptoms, respectively. Less negative *δ*^13^C values suggest higher water-use efficiency and thus higher drought avoidance capability. Significance: ^*p* < 0.1 *p < 0.05, **p < 0.01, ***p < 0.001, n.s. not significant. (*b*) The MAT transfer distance (ΔMAT) was calculated as the difference in MAT between a test site and a population-origin location. Positive (negative) values indicate MAT_garden_ > MAT_population_ (MAT_garden_ < MAT_population_), respectively. Filled black circles with 95% CIs were plotted for each population in each test site. Population mean across common gardens and common-garden mean across populations are portrayed by different shapes. Quadratic regression is plotted on the graph with adjusted pseudo-*R*^2^ estimated. (*c*) We, respectively, classified source populations and test sites by MAT to cold versus warm groups, as noted in figure 1.

Correlation analysis revealed relationships between focal traits and climatic characteristics. There was an intermediate, positive correlation between height and *δ*^13^C (Pearson's *r* = 0.302, *p* < 0.05; electronic supplementary material, figure S2), whereas correlations between pest suitability versus height or pest suitability versus *δ*^13^C were low and not significant (all |*r*| < 0.05, *p* > 0.05; electronic supplementary material, figure S2). Height and *δ*^13^C exhibited a high relationship with mean annual temperature (MAT) of population origin (*r* = 0.81 and 0.95, respectively; significance for height with *p* = 0.05 and *δ*^13^C with *p* < 0.05; figure 2*a*), whereas the correlation of WGR severity and MPB suitability with MAT was low and intermediate, respectively (*r* = −0.1 and 0.65, not significant at *p* > 0.05; figure 2*a*). Likewise, there were high correlations of height and *δ*^13^C with mean annual precipitation (MAP) of origin (*r* = 0.67 and 0.79, respectively; not significant at *p* > 0.05); nonetheless, MAP correlation with WGR was intermediate (*r* = 0.75) contrasting with its low correlation with MPB (*r* = −0.1), albeit not significant (*p* > 0.05) for both. These correlation patterns revealed that height and *δ*^13^C displayed a co-gradient variation with MAT, and that there existed a negative trend between WGR and MAT, and MPB and MAP; a positive trend between MPB and MAT, and WGR and MAP (figure 2*a*). Populations stemmed from warm sites had greater height and *δ*^13^C in test sites compared to those from cold sites of origin (figure 2*a*). We found that height and *δ*^13^C were expressed differently in test sites with the highest mean values in warm sites (i.e. JUDY and VIRG versus SWAN and TIME; electronic supplementary material, figure S3). Both traits in test sites were greater for populations transferred from warm locations (figure 2*b*); when populations were transferred to warmer test sites, both traits were enhanced (figure 2*b*). Meanwhile, WGR severity increased when populations were transferred to warmer sites (figure 2*c*); however, cold-origin populations were more prone to WGR infection than warm-origin populations (figure 2*c*). On the contrary, warm-origin populations were more conducive to MPB attack than cold-origin populations in both warm and cold test sites (figure 2*c*). It is noteworthy that all test sites were relatively warmer and drier than most of the population-origin sites ( figure 2*b* horizontal axis range and figure 1 legend).

### (b) Evolutionary potential measures

**Figure 3.**
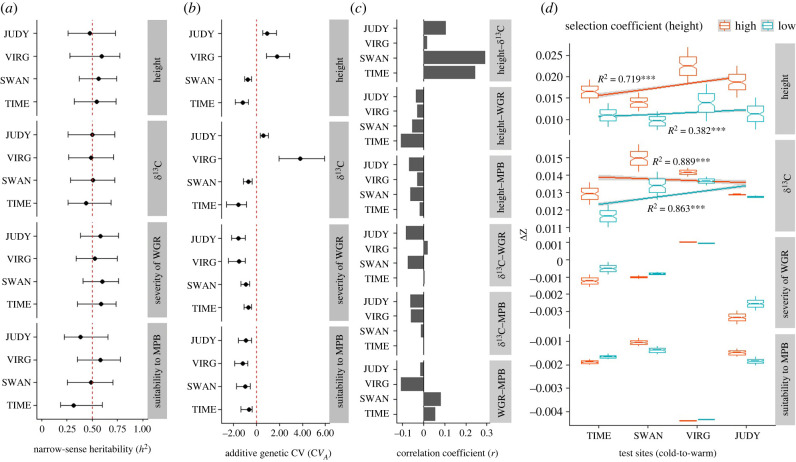
Narrow-sense heritability (*h*^2^), additive genetic coefficient of variance (C*V*_A_), between-trait correlation and predicted evolutionary response to selection (ΔZ) for focal traits of *Pinus contorta* in each test site over one generation. (*a*) The *h*^2^ values with the proportion of phenotypic variance contributed by additive genetic variance were estimated from phenotypic data. (*b*) We used phenotypic data to estimate *V*_A_ for the C*V*_A_ calculation. C*V*_A_ is dimensionless. All traits were scaled. For visualization convenience, C*V*_A_ for MPB was multiplied by 10^4^ on the graph. (*c*) The Pearson's correlation coefficients were calculated for all pairs of traits measured in each test site. (*d*) We used the posterior means over 10 000 Markov chain Monte Carlo samples in calculating the predicted response to selection (ΔZ) for each focal trait in four test sites. ΔZ was estimated based on the multi-variate breeder's equation (ΔZ = G*β*). The 95% CIs were generated by using two sets of *β*-values for height: 50% lower or higher selection gradients than in *δ*^13^C.

We compared evolutionary potential of focal traits using two genetic measures, *h*^2^ and C*V*_A_. Estimates of *h*^2^ of each trait did not differ substantially between test sites (figure 3*a*). Average point estimates of *h*^2^ were about 0.5 for these traits (figure 3*a*), indicating significant additive genetic variation and that these traits are under strong genetic control. Metrics of C*V*_A_ in height and *δ*^13^C were remarkedly higher in JUDY and VIRG (figure 3*b*). By contrast, C*V*_A_ had no noticeable difference in WGR or MPB between test sites, and C*V*_A_ was close to zero (10 × 10^−4^) for MPB compared to about −1 for WGR (scaled for showing in figure 3*b*).

### (c) Estimation for phenotypic selection and response to selection

By performing selection analysis via height as a fitness proxy in a univariate manner, we identified selection patterns for each trait in each test site. The form of selection in *δ*^13^C and pest suitability differed depending on test sites (table 1). The *δ*^13^C trait was under directional selection (*p* < 0.0001) in the two cold sites, TIME and SWAN (table 1), and under both directional and diversifying selection (*p* < 0.05) in a warm site, JUDY (table 1). MPB suitability was under divergent selection at SWAN and JUDY (*p* < 0.05; table 1), whereas WGR severity was under directional selection at TIME and divergent selection at SWAN (*p* < 0.05; table 1). Moreover, these selection patterns were reflected by the strength of correlation between height and another focal trait studied. Specifically, height-δ^13^C correlation was higher in TIME and SWAN than in JUDY and VIRG (*r* = 0.25 versus 0.08; figure 3*c*); there was a higher correlation between height and WGR in TIME compared to the other sites (*r* = −0.12 versus −0.04; figure 3*c*). In addition, we observed that relatively low correlations between height and *δ*^13^C in the two warm sites were caused by populations responding differently in height and *δ*^13^C at warm sites compared to a consistent positive pattern at cold sites (electronic supplementary material, figure S4).

Further, a multi-variate analysis consistently showed increased response to selection for height and *δ*^13^C in warm environments after assuming that the selection gradient in height was lower than that in *δ*^13^C (figure 3*d*). However, given a higher selection gradient in height relative to *δ*^13^C, response to selection for *δ*^13^C was lower in warm than cold sites, whereas height remained more selected for in warm sites with less selective intensity compared with the previous scenario (*r* = 0.62 versus 0.85, *p* < 0.0001; figure 3*d*). With regard to pest suitability, the overall response to selection was lower than height or *δ*^13^C by one order of magnitude (figure 3*d*). The two cold test sites had relatively high responses to selection for both WGR and MPB, whereas a warm, more rainfall climate (e.g. VIRG) led to the highest (lowest) response to selection for WGR (MPB), respectively (figure 3*d*). Similarly, a warm, less rainfall climate (e.g. JUDY) resulted in the lowest response to selection for WGR but a relative high response to selection for MPB (figure 3*d*). Moreover, we observed only one positive response to selection for a pest suitability—WGR at JUDY (figure 3*d*). These patterns in warm climate were in line with a negative correlation between WGR and MPB at VIRG and JUDY (figure 3*c*). Furthermore, the efficacy of selection based on *G*-matrices indicated that VIRG and JUDY had a smaller correlation between height and *δ*^13^C (0.01–0.03) than SWAN and TIME (0.09–0.11) (electronic supplementary material, figure S5). Meanwhile, autocorrelation for these two traits was higher in VIRG and JUDY than in SWAN and TIME (0.51–0.59 versus 0.3–0.38 and 0.43–0.44 versus 0.34–0.38 for these two traits, respectively) (electronic supplementary material, figure S5).

## 4. Discussion

We showed how warming affects the evolution of height growth versus resistance traits in a uni- and multi-dimensional trait space by planting seed-source populations of lodgepole pine in four test sites, mimicking future *in situ* climate change scenarios over time. The selected populations were based in the species range edge, possibly under the greatest exposure to climatic change. These common-garden studies revealed that warming would promote evolutionary response to the selection of both height and *δ*^13^C, and affect host suitability to pests depending on precipitation. Due to fluctuating weak response to the selection of pest suitability, there was a limited evolutionary influence of pest suitability on height and *δ*^13^C response. The significance of the work accentuates weak selection with high variability in pest suitability, according with subtle ecological association between warm climate (warm origin or test site) and high pest attacks; moreover, biotic pressures from pests have a limited impact on the evolution of height growth and *δ*^13^C.

### (a) Do height growth and drought avoidance always possess a synergistic relationship and high evolutionary potential under warming?

It has been widely accepted for the use of *δ*^13^C as an indicator of the intensity of drought exposure in plants (e.g. [37,38]). This study showed that fast-growing populations had greater xylem hydraulic conductance (i.e. high *δ*^13^C) in warm test site, indicating the importance of maintaining water conductance to growth in warmer conditions. While *δ*^13^C indicates drought avoidance by measuring reduced water loss—a water-saving strategy, drought avoidance also involves enhanced water uptake from roots—a water-spending strategy [39]. Trees could rely on resource investment in rooting depth to increase access to deep soil water to withstand drought stress [40,41]. Our investigation of drought avoidance strategies inferred from *δ*^13^C could be improved by the further investigation into the below-ground determinants of plant water relations using combinations of hydraulic traits such as P_50_ (i.e. the water potential at which 50% of hydraulic conductivity is lost) and water potential at stomatal closure or turgor loss (e.g. [42,43]). Combining multiple interlinked, yet distinct, aspects of plant water relations can better quantify water-use strategies based on interactions between plant traits and environmental conditions. Moreover, considering other drought adaptive strategies including drought escape (e.g. flowering or pollination time), tolerance (e.g. osmotic potential) and resilience (e.g. dendrochronological measure indicating recovery capacity after drought) [22] allows for a better understanding whole-plant drought strategies and their relationship with plant growth and pest resistance.

We observed that height and *δ*^13^C differed in populations and test sites. Higher positive sensitivity to temperature in both traits at relatively warmer sites today tells us that trees at relatively cooler sites may anticipate more rapid growth and greater *δ*^13^C in a warmer future. Moreover, high additive genetic variation or heritability suggests that directional selection could increase adaptability to novel climatic scenarios. Consistently, we found that both height and *δ*^13^C had greater evolutionary potential under a warmer exposure based on C*V*_A_. It is worthwhile to note that the other metric used—*h*^2^ may not reflect the true potential of adaptive evolution due to environmental variation under natural conditions greatly affecting phenotypic traits and fitness, leading to a possibility of small or no significant change in *h*^2^ even when *V*_A_ is high or greatly alters [44,45]. An instance in *P. sylvestris* also showed that progeny derived from warmer climates outperformed local seed sources in ‘cold’ locations, and local seeds grew best locally only in very warm source locations [46]. In addition to adaptive evolution, lodgepole pine hybridization with jack pine (*P. banksiana* Lamb) at our study region has been found to enable an expansion of range limits eastward [47], providing another evolutionary avenue for these pine range-margin populations to enhance genetic variation and adapt to changing climates.

### (b) Selection and evolution of host suitability to pests under warming

We found a positive or negative trend along a thermal cline for two constitutive defences against a phytopathogen and an insect herbivore, respectively. Populations from warm versus cold environments had an inverse pattern of these defence traits in test sites. This indicates that tree suitability to different species of pests varies under different environmental conditions. Nonetheless, this study used height growth as a proxy for fitness, which might limit our inference about the evolution of traits including pest suitability under climate change. There are three main components of plant fitness including growth, reproduction and survival [48]. Central to these components is metabolism, providing the carbon necessary for allocation to various structures and functions. However, natural selection that operates on pest suitability and functional traits is likely more by directly affecting tree survival and reproduction than through their relationship with tree growth. Although trees with slower growth rates are found to be more likely to die than faster growing counterparts (e.g. [49]), a first-order constraint on plant growth is photosynthetic capacity in assimilating energy and matter.

Further, this study investigated trait patterns in association with two climatic drivers—MAT and MAP. Other than climate, edaphic conditions could be another important selective force driving the evolution of growth and resistance traits [50]. The test site JUDY had a more acidic brunisolic soil with a pH of 3.9, compared to a luvisolic soil with a pH of 5.5 in.the other test sites (electronic supplementary material, table S1). The difference in soil texture may contribute to the disparity observed in response to the selection of pest suitability in JUDY versus the other test sites.

Previous studies confirmed that pine populations grown in optimal growing conditions had higher susceptibility to pests than in less favourable conditions [51]. This study demonstrated that selection in pest suitability was much weaker than *δ*^13^C and varied greatly in two warm sites with different rainfall, suggesting fluctuating weak selection in pest suitability. This selection pattern in pest suitability could be interpreted by pine life-history characteristics. *Pinus contorta* commences reproduction at around 10 ± 5 years old [52,53], whereas MPB doesn't typically attack trees until they are much older, that is, greater than 60 years [54,55]. *P. contorta* produces serotinous cones with viability for up to 10–15 years after the tree has been killed [56,57]. This chronological discrepancy provides an extended period during which trees that will be ultimately killed by MPB can still grow and reproduce. Furthermore, the thickness and constituents (e.g. nutrients and toxic secondary compounds) of phloem, which are usually positively correlated with tree age and size, are main direct factors affecting host suitability to bark beetles or other phloem-feeding insects [7,58]. In addition, conifer defences against bark beetles are strongly mediated by environmental stress [16,59], which increases uncertainties in defence selection.

### (c) Evolutionary interactions of height growth, drought avoidance and pest suitability

This study revealed that climate strongly influenced the pattern of selection in *δ*^13^C and host suitability to pests, albeit overall weak for pest suitability. As opposed to selection on isolated traits, multi-variate analysis assumes that selection acting on one trait will produce an evolutionary response in other genetically correlated traits, even though selection does not act directly on them. Prediction of evolutionary changes in multiple traits relies on the form and magnitude of selection in height growth and *δ*^13^C, and on historical influences from pest attacks (e.g. [60]). We found that evolutionary response to selection was strongest for height in warmer sites, in accordance with its high adaptability and evolutionary potential. Warming could also promote response to selection in *δ*^13^C if it was under stronger selection than height growth. If selection is stronger in height than *δ*^13^C, then we could expect that a great extent of warming would likely select against high *δ*^13^C. These particular results suggest that height growth is always selected for to maintain a direct performance gain, but its strength of selection affects evolutionary changes in *δ*^13^C. Furthermore, higher response to selection of height and *δ*^13^C in warm sites may be modulated by populations responding differently to warm conditions (i.e. a strong selective pressure) and higher selective efficiency under warming.

In addition, we demonstrated that under warming conditions, precipitation significantly affected response to selection in pest suitability, indicating that selection is likely to be affected by warm temperature and rainfall interactions. As such, we could expect different patterns of selection under dry versus humid hot or through temperature and precipitation interactions. Pest outbreaks are highly contingent on climate with contrasting impacts for dry hot versus humid droughts and plant defences are highly variable across a gradient of the environment [61], indicating possibly variable selection over space and time.

In conclusion, this study provides evidence that rising temperatures are beneficial to adaptive evolution in height growth and *δ*^13^C (drought avoidance), resulting in taller and more drought-tolerant trees, and that biotic pressures from pest attacks have a limited influence on evolutionary response to the selection of height growth and *δ*^13^C. Nonetheless, as trees are sessile organisms with a long-life cycle of multiple decades or even centuries but most pest species have seasonal migration in an annual cycle [62], trees attacked by pests are determined largely by pest behaviour and tree host–environment–pest interactions. As climate changes, we could expect shifts in evolutionary response to the selection of growth and drought avoidance towards high values without too much evolutionary constraints by pest suitability. Great impacts of pest suitability on growth or fitness would be generated primarily at the ecological level possibly by a sudden massive pest attack further exacerbated through an interaction with drought spells.

## Data Availability

Data and modelling code supporting this paper are available from Dryad Digital Repository [63]. Electronic supplementary material is available online [64].
